# A Comparative Study of the Assessment of the Accuracy of Willems’ and Demirjian’s Methods in Dental Age Estimation of the Saudi Arabian Population

**DOI:** 10.7759/cureus.50128

**Published:** 2023-12-07

**Authors:** Mohammed Alrashidi, Anas Al-Moshiqah, Shaul Hameed Kolarkodi, Faraj Alotaiby

**Affiliations:** 1 Dental Clinics, Medical City, College of Dentistry, Qassim University, Buraydah, SAU; 2 Dentistry, Qassim University, Buraydah, SAU; 3 Maxillofacial Surgery and Diagnostic Sciences, College of Dentistry, Qassim University, Buraydah, SAU

**Keywords:** saudi arabia, willems’ methods, orthopantomography, demirjian method, age estimation

## Abstract

Background: The notion that "age is just a number" is sometimes expressed colloquially; however, within the domains of medicine and dentistry, age is not only a numerical value. Rather, it has significant importance in several facets of diagnosis and treatment planning. Demonstrating one's identity is necessary not only in the aftermath of natural calamities or mishaps but also in the context of forensic examination of living persons. Age assessment is a very important tool in forensic odontology and plays a vital role in various fields, including medico-legal matters. The primary focus of the current investigation is to assess the precision and correctness of the data and reliability of Demirjian's method (DM) and Willems' method (WM) for age estimation (AE) among the population of Saudi Arabia.

Methodology: This research was conducted on 300 children from Saudi Arabia, aged seven to 13 years, including both males and females. The assessment of orthopantomography images involved the utilization of both Demirjian's and Willems' methodologies for ascertaining dental age. This determined dental age was then meticulously juxtaposed with the chronological age (CA) of every participant. The data collected underwent a comprehensive statistical analysis, which encompassed the application of the paired t-test.

Results: By using both methodologies, it was discovered that the estimated age (EA) exhibited higher values in both men and females compared to the CA. Both Willems' and Demirjian's approaches yielded significantly different results in terms of statistical significance (p = 0.000 and p = 0.000, respectively), as shown by the comparison.

Conclusion: When comparing Willems' technique to Demirjian's method, it was found that the population under research showed somewhat greater accuracy levels for AE. However, it is important to note that the disparity between these two approaches was relatively small. Consequently, it is imperative to emphasize the necessity for additional research involving a larger sample size to establish the validation of a more region-specific AE method tailored specifically for the Saudi Arabian population.

## Introduction

The precise determination of age, often referred to as accurate age estimate (AE), has significant importance in the field of forensics, with applications extending across several disciplines, including pediatric dentistry, anthropology, and archaeology [1,2]. Accurate age assessment plays a pivotal role in various medico-legal sectors, proving essential in forensic contexts such as estimating the age of deceased individuals following natural disasters and catastrophes, determining the age of illegal immigrants, establishing legal liability, aiding in juvenile criminal cases, and supporting adoption proceedings [3-6]. This comprehensive approach to age determination is crucial in ensuring justice and addressing a wide range of legal and humanitarian concerns.

Numerous age assessment techniques have been proposed over time, but only a select few have gained widespread recognition. These methods are founded on various criteria, including histological factors, while others rely on radiographic data. Radiographic approaches offer a distinct advantage due to their non-invasive nature, enabling their use in both living individuals and post-mortem examinations. In the process of determining a patient's dental age, several radiographic techniques are used. Some of these techniques include Demirjian's method (DM), Kvaal's method, Willems' method (WM), Nolla's method, and a great deal of others [7]. Among these radiographic methods, DM and WM stand out as the most commonly utilized [8,9]. However, it is imperative to verify the applicability of DM and WM, initially developed using research populations from France, Canada, and Belgium to the Saudi Arabian population. DM offers a notable advantage by providing a set of objective criteria for the characterization of different stages of tooth development. This methodology has gained widespread recognition and has become the predominant approach for assessing dental age [8-10]. Previous research employing DM on diverse populations has documented variations in dental development, encompassing both accelerated and decelerated growth patterns [11-13].

In 2005, Willems et al. [9] introduced significant modifications to the DM by devising new tables that enabled the direct expression of a maturity score in years. The modification described in the statement removed the need to translate the maturity score into a dental age, streamlining the process while still preserving the intrinsic benefits of DM [13]. The importance of age estimation for children has been on the rise in Saudi Arabia due to various factors, including illicit human trafficking, emigration, deportation procedures, cases involving child exploitation, adoption of children without birth certificates, and legal considerations [14-19]. The legal system in Saudi Arabia employs distinct judicial measures for individuals aged seven years and older, differing from those applied to those below this age threshold [14].

Research in Saudi Arabia has focused on determining an accurate AE using DM in individuals from the Middle and Western regions [15]. Nevertheless, there is a lack of comprehensive research on the accuracy of DM in estimating the age of children in Saudi Arabia, and the need for a specific reference technique designed for the Saudi Arabian population is still questionable [14]. Therefore, the major objective of this research was to validate DM and WM in a group of Saudi teenagers, with the end goal of developing a Willems' technique that is tailored to the demographics of Saudi Arabia (Willems SA). Additionally, the study sought to compare the prognostic precision of these two methodologies for age estimation within this specific population.

## Materials and methods

This cross-sectional study was performed at the College of Dentistry, Qassim University. The sample size for the study was determined based on previously published studies, considering the statistical power. The determination of the needed sample size was conducted based on the effect size, leading to the conclusion that a total of 500 individuals were necessary to reject the null hypothesis. A comprehensive collection of orthopantomographic data, consisting of 500 children, was retrieved from the dental records of dental institutions and private clinics across Saudi Arabia. The age range of the participants in the study ranged from seven to 13 years, with an average age of 9.31 years for males and 9.26 years for females. Inclusion criteria for the radiographs included high-quality panoramic images with no blurring or artifacts around the tooth roots and clear documentation of both the birth date and the date of the radiograph. Exclusion criteria encompassed systemic disorders, dental malformations, nutritional and endocrine diseases, birth deformities, head rotation causing variations in tooth size between quadrants, and the absence of one or more permanent mandibular teeth.

Two researchers, each of whom had undergone training and calibration to provide a reliable and accurate age assessment utilizing orthopantomography, were responsible for carrying out the study. This training was conducted in collaboration with the Department of Radiology, and the inter-examiner agreement was documented. After acquiring the radiographs, a trained examiner conducted the assessments under the supervision of an oral radiologist.

**Figure 1:**
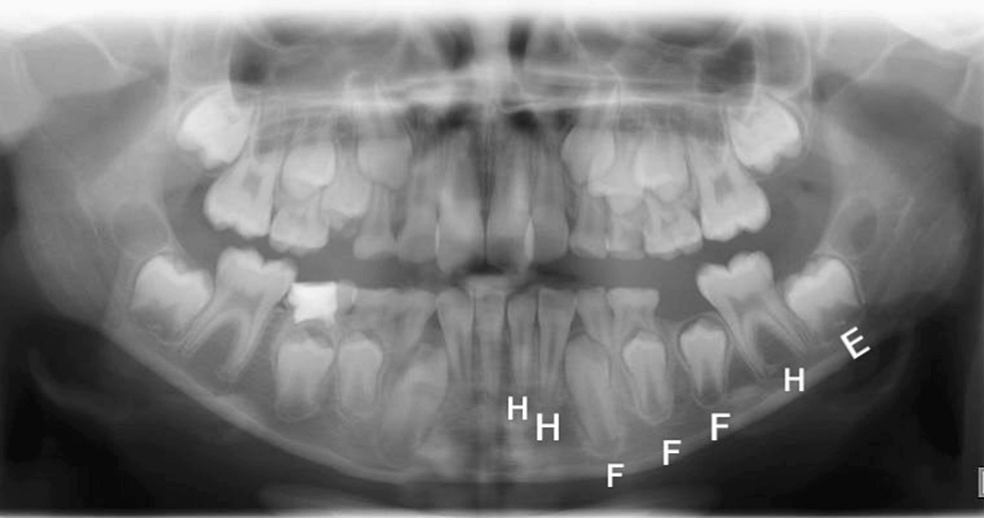
Panoramic radiograph with stages of calcification from E to H

Both the approaches outlined by Demirjian et al. in 1973 and those outlined by Willems et al. in 2001 were used in the analysis of the radiographs. The acquired data included a coordinated pair [8,9], and the evaluation procedure was restricted to the seven teeth located on the left side of the jaw, with the exception of the third molar. The first assessment included categorizing the calcification state of each tooth into categories ranging from "E" to "H" (Figure 1). Following the criteria established by DM and WM [8,9], the scores of each participant were afterward transformed into a dental age, taking into account their gender.

The current research used the “weighted analysis of variance” approach proposed by the Willems BC model to calculate dental maturity scores for all teeth in both male and female participants [8]. To undertake a rigorous examination of the disparity between estimated age (EA) and recorded chronological age (CA), we classified the data according to gender and age cohorts, afterward doing a comparison analysis.

The data gathered were inputted, arranged, and formatted using Microsoft Excel version 2003 (Microsoft Corporation, Redmond, WA). The statistical analysis was conducted via IBM SPSS version 25 (IBM Corp., Armonk, NY). The data are presented as the mean plus or minus the standard deviation. To assess the statistical significance, a paired t-test was used, with a p-value threshold of 0.05 indicating potential statistical significance. The agreement among examiners was assessed using the Kappa statistic test, whereas the association between CA and DM, as well as WM, was investigated using the Pearson correlation test. A p-value below the threshold of 0.05 is regarded as statistically significant.

## Results

The present research was undertaken at the College of Dentistry, Qassim University, comprising a sample of 500 people aged between seven and 13 years. The major purpose was to examine the accuracy of DM, WM, and EA in relation to CA. Before commencing the study, both examiners received training from the Department of Radiology to ensure precise age determination using DM and WM. Inter-examiner agreement was assessed using kappa statistics, which yielded a coefficient of 0.82, indicating a high level of agreement.

To evaluate the statistical significance of the disparity between the control group (CA) and the experimental group (EA), a paired t-test was used. Notably, both male and female samples underwent assessment using two distinct methodologies. The relevant data for analysis and comparison are presented in Table 1.

**Table 1:** Information about age estimate techniques for individuals of both male and female genders

Gender	Group	N	Mean ± SD	p-value
Male	Chronological age	300	9.31 ± 2.79	0.00
	Demirjian's method	300	9.47 ± 2.09	
	Chronological age	300	9.31 ± 2.79	0.00
	Willems' method	300	9.94 ± 2.69	
Females	Chronological age	200	9.26 ± 2.78	0.00
	Demirjian's method	200	9.49 ± 2.15	
	Chronological age	200	9.26 ± 2.78	0.00
	Willems' method	200	9.62 ± 2.85	

The table provides information about age estimate techniques for individuals of both male and female genders. Regarding men, the average CA was calculated to be 9.31 ± 2.79, but the average age estimations derived from DM and WM were 9.47 ± 2.09 and 9.94 ± 2.69, respectively. The p-values for comparing these different age estimation methods were all found to be 0.00, signifying statistically significant differences between the methods. Notably, both DM and WM yielded mean age estimates that were significantly higher than those obtained using CA (p < 0.05).

For females, the mean CA was estimated to be 9.26 ± 2.78, while the mean age estimates obtained through DM and WM were 9.49 ± 2.15 and 9.62 ± 2.85, respectively. The p-values obtained for comparing these various age estimation methods were also 0.00, indicating statistically significant differences among the methods. In the case of females as well, the mean age estimates derived from DM and WM were notably higher than those obtained from CA, with a p-value less than 0.05.

The research used Pearson correlation analysis to examine the association between two age estimate methodologies and CA. A robust positive connection was identified between CA and the two distinct age estimate methods in both male and female cohorts. Furthermore, a statistically significant association was observed, as shown by a p-value below the threshold of 0.05. The pertinent data are shown in Table 2.

**Table 2:** Pearson correlation of chronologic age with different age estimation methods

Chronological age	Male		Female	
	Demirjian's method	Willems' method	Demirjian's method	Willems' method
Pearson correlation	0.312	-0.363	0.342	0.259
Sig. (2-tailed)	0.002	0.000	0.000	0.009
N	300	300	200	200

## Discussion

In 1837, Edwin Saunders highlighted the significance of teeth in age estimation by publishing an article titled "Teeth: A Test of Age" [16]. Subsequently, other methodologies for estimating age have been investigated; nevertheless, a widely acknowledged approach has yet to be established [17]. The technique developed by Demirjian is often used for age estimates owing to its ease of use and directness [18]. Previous studies using DM in different populations have yielded varying results [19-21]. Hagg and Matsson's study in 1985 found that DM demonstrated high accuracy and precision when applied to children in Sweden [17].

The obtained results from the current study reveal noteworthy insights into the age estimation methods, particularly in the context of gender-based variations. For males, both DM and WM exhibit statistically significant differences when compared to CA (p < 0.05). While DM yields a slightly higher mean age than CA, WM tends to overestimate age to a greater extent. This disparity suggests that, in the male population, the selected dental age estimation methods may not consistently align with the actual CA. Similarly, in the female group, both DM and WM demonstrate statistically significant differences from CA (p < 0.05). Notably, DM tends to overestimate age, with a higher mean compared to CA, while WM also exhibits a similar trend but to a lesser extent. These findings underscore the importance of gender-specific considerations in the application of dental age estimation methods, cautioning against a one-size-fits-all approach.

In a study conducted by Esan et al., it was revealed that there was a tendency to overestimate age in both male and female groups until the age of 16 years [13]. On average, men overestimated their age by 0.62 years, while girls overestimated by 0.74 years. The study conducted by Koshy and Tandon in 1998 showed that there was an overestimation of 3.04 years in men and 2.82 years in females among young individuals from South India [20]. In a recent research by Al-Emran et al., an investigation was carried out on a cohort of children in Saudi Arabia [21]. The study's findings revealed a little disparity between dental age and CA. Specifically, the results indicated that men exhibited an average discrepancy of 0.3 years, while females displayed an average difference of 0.4 years. In a study conducted by Baghdadi et al., it was observed that there was a mean difference of 0.77 ± 0.85 years in males and 0.85 ± 0.79 years in females [1]. Ahmed conducted a research investigation on the phenomenon of dental age advancement in a sample of children aged three to 15 years [22,23]. The findings revealed that males exhibited an average dental age advancement of 0.57 ± 1.48 years, while females demonstrated an average advancement of 0.44 ± 1.66 years. The amalgamation of these diverse studies underscores the variability in age estimation methodologies across different populations, emphasizing the need for region-specific considerations and the potential impact of gender on the accuracy of such methods [22-24]. These collective findings contribute valuable insights to the ongoing discourse surrounding dental age estimation, supporting the argument for nuanced approaches tailored to specific demographic characteristics [25,26].

The findings of the study indicated that there was a tendency to overestimate the perceived age of persons, with men being overestimated by an average of 0.63 years and females by an average of 0.36 years. The efficacy of the Willems technique is significantly accurate when used in the Turkish adolescent population. The study conducted by Altan et al. in Turkey yielded findings indicating that females exhibited greater levels of accuracy in working memory (WM) tasks when compared to males [27,22]. Numerous studies have shown a tendency to overestimate dental age when using the wrist and hand maturation method concerning CA [28-30]. Cherian et al. conducted research in Malaysia to investigate age estimate accuracy among individuals aged six to 15 years. The findings revealed that males displayed a mean age overestimation of 0.04 ± 1.08 years, while females had a mean age overestimation of 0.03 ± 1.18 years [19]. Furthermore, the study's results suggest that the methodology used by Willems yields more accurate estimations of age in females than in males. This discrepancy may be attributed to disparities in the temporal progression of development between the two sexes, which aligns with the reported phenomenon of accelerated skeletal maturity in females.

Future research could explore the impact of genetic and environmental factors on dental development, contributing to a more nuanced understanding of age estimation in diverse populations. Additionally, investigating the influence of cultural and nutritional variables on dental maturity may enhance the accuracy of age estimation methods [31]. The development of a region-specific age estimation model tailored to the unique characteristics of the Saudi Arabian population remains a promising avenue for future research. While the current study adds valuable insights, continuous efforts to refine age estimation methods and address specific population considerations will contribute to advancements in forensic odontology and related fields.

## Conclusions

In conclusion, the current study reveals that, when it comes to AE, DM tends to exhibit slightly higher accuracy than WM for both male and female participants. However, it is important to note that the differences in average age estimations among the various methods are minimal, suggesting that the practical significance of these variations may be negligible. Additionally, it should be emphasized that the generalizability of our findings to other populations or contexts may be limited, and further research may be required to validate the accuracy of these methods in predicting age.
